# Reference Curves of Selected Circumferential Parameters for Czech Children Aged 6 to 11 Years

**DOI:** 10.3390/children8100908

**Published:** 2021-10-13

**Authors:** Jancikova Vendula, Pridalova Miroslava, Kaplanova Tereza

**Affiliations:** Department of Natural Sciences in Kinanthropology, Faculty of Physical Culture, Palacký University, 771 11 Olomouc, Czech Republic; miroslava.pridalova@upol.cz (P.M.); tereza.kaplanova@upol.cz (K.T.)

**Keywords:** waist circumference, abdomen circumference, gluteal circumference, anthropometry, percentiles, children

## Abstract

(1) Background/objective: the globally discussed current issue is the increasing body weight of the population. This trend is observed in all age categories. Pediatricians and anthropologists use BMI percentile curves to determine the optimal body weight of children, based on which the child is categorized in the category of underweight, normal weight, overweight, or obese. Medical or anthropological examinations also use some methods to determine the amount of body fat. In addition to evaluating amounts of body fat, it is important to assess its distribution. The distribution of fat in the body, especially in terms of disproportionate distribution, is a risk factor for health complications, especially in terms of metabolic and health risk. Part of monitoring children’s growth is also measuring and evaluating circumferential parameters, such as abdominal circumference, gluteal circumference, and waist circumference. This study aimed to define age- and gender-specific reference curves for waist circumference (WC), abdominal circumference (AbC), and gluteal circumference (GC) in Czech children. (2) Methods: data on children’s circumferential parameters were collected via anthropometric measurements. The research sample consisted of 2093 children aged 6–11 years (boys, *n* = 1008; girls, *n* = 1085). Only children with parental informed consent were included. The statistical analysis was performed separately by age and gender using SPSS v. 22. Anthropometric data were summarized by mean and standard deviation. The percentile curves of WC, AbC, and GC were calculated (P3, P10, P25, P50, P75, P90, P97) in R 3.4.2 software (R Foundation for Statistical Computing, Vienna, Austria) using the gamlss package. (3) Results and conclusions: the study developed age- and gender-specific percentile curves of WC, AbC, and GC for Czech children aged 6–11 years. All parameters increased with age in both boys and girls. Generally, the boys had higher WC and AbC than did girls, but girls had higher GC than did boys. Female and male median WC percentiles (M) increased from the age of 6. Both girls’ and boys’ median percentiles showed a continuous increase. We found similar trends in the median GC and AbC percentile curves. All percentile curves showed similar trends in both sexes, but the 90th and 97th WC percentiles in boys were exceptions: from the age of 10, they exceeded the values of girls, reaching their peak at the age of 11 followed by a decrease in the case of the 97th percentile and a plateau in the case of the 90th percentile. This study serves as a reference to enrich the methods of evaluation of somatic and medical status in Czech children. Up–to–date percentile curves would be a practical addition to the BMI percentiles for the screening and evaluation of overweight and obese conditions and the related risks of abdominal obesity in the pediatric population.

## 1. Background

Pediatric obesity is a public health concern. The worldwide prevalence of overweight and obese children and adolescents has reached worrying levels [1]. The Czech Republic is not an exception [2].

Anthropologists and physicians, on the basis of standard anthropometric examination, measure and then evaluate somatic parameters, which provide important information about the growth status in children and adolescents. Anthropometry is the most used and preferred clinical tool to follow the normal growth of a child [3]. Anthropometric measures might also be helpful in the prediction of non-communicable disease risk factors from childhood [4,5,6]. Anthropometric examination usually evaluates the body weight and, if the body composition is analyzed, the amount of body fat. However, its distribution on the body must also be observed, especially in terms of its disproportionate distribution, which is a risk factor for health complications, especially metabolic and cardiovascular [7].

In the Czech Republic, pediatricians most often use percentile BMI curves, which were constructed on the basis of the results of the National Anthropological Survey (NAS) from 1991 and 2001, to assess the child’s weight status [2]. At the population level, a percentile curve is a useful tool for monitoring and screening obesity among school-aged children and adolescents [8].

Body mass index (BMI), calculated as weight in kg/(height in metres)^2^, is perhaps the most widely used surrogate for excessive adiposity in humans. However, BMI has some disadvantages. Recent research has demonstrated that BMI is a limited indicator of pediatric metabolic risk because of its limited specificity in children [9]. BMI alone cannot reflect fat distribution very comprehensively, such as abdominal fat deposits [10]. Abdominal obesity, which is the deposition of visceral fat, is associated with a higher risk of metabolic complications, such as hyperlipidaemia and diabetes [11]. Hypertension can be another health consequence of obesity. Gluteal circumference (GC), which is associated with the amount of visceral fat, may be useful in detection [12]. 

An additional anthropometric indicator is waist circumference (WC), which is a relatively simple and convenient measure and can be readily used to estimate the accumulation of abdominal fat [13]. WC has emerged as an index of pediatric adiposity that predicts fat mass as well as or better than BMI [14,15]. WC has proven to be effective for estimating total adiposity and has been validated against various other body composition methods, including, for example, bioelectrical impedance (BIA) [16]. BIA is valid and reliable; it provides conclusive information on the representation of individual body fractions, within the basic population spectrum [17]. However, in the Czech Republic, BIA is definitely not a standard pediatric measure.

In this study, we aimed to construct age- and gender-related percentile reference curves for WC, GC, and abdominal circumference (AbC), because it is necessary to update them. In the case of WC, there are no existing reference data for Czech children. Up-to-date percentile curves would be a practical addition to the BMI percentiles for the screening and evaluation of related risks of being overweight and obese in the pediatric population. 

## 2. Methods

### 2.1. Subjects and Anthropometric Measurements

The data used in this study were drawn from research conducted at 23 selected primary schools in five regions in the Czech Republic. The research sample consisted of 2093 apparently healthy children aged 6–11 years (boys, *n* = 1008; girls, *n* = 1085). In accordance with the ethical principles of the Declaration of Helsinki, the parents and all children accepted and signed the informed consent form [18].

Data on children’s waist circumference were collected via anthropometric measurements by a trained technician using a tape measure at just above the uppermost lateral border of the right iliac crest at the end of a normal expiration. Gluteal circumference was measured at the greater trochanters at the widest part of the hips. Abdominal circumference was measured approximately at the umbilicus level, where the abdomen is most arched. Accuracy must be observed when determining circumferential parameters. The probands were measured by the same experienced technician. The circumferential parameters were measured with the accuracy of 0.5 cm. We followed exactly the instructions for standard anthropometric measurement [18].

The measurements were taken at the primary schools in the morning under standard conditions while maintaining a high level of hygiene. 

### 2.2. Statistical Analysis and Centile Curves

The research data were sorted out using an MS Excel spreadsheet and were statistically processed by IBM SPSS Statistics for Windows, Version 22.0 (IBM Corp., New York, NY, USA). The statistical analysis was performed separately by age and sex. Anthropometric data were summarized as mean and standard deviation. The statistical significance of differences between genders in body height, body weight, waist circumference, abdominal circumference, and gluteal circumference was assessed by the mean compared test. The level of statistical significance was selected as α = 0.05.

The percentile curves of WC, AbC, and GC were calculated (P3, P10, P25, P50, P75, P90, P97) in R 3.4.2 software (R Foundation for Statistical Computing, Vienna, Austria) with the use of a gamlss package.

## 3. Results

The study included a sample of 2093 elementary school children aged 6–11 years. The male-to-female ratio was 0.93: 48.2% boys and 51.8% girls. 

The descriptive characteristics of the height, weight, WC, AbC, and GC by gender and age are summarized in Table 1. All parameters increased with age in both boys and girls. Generally, the boys had higher WC and AbC than did girls, but the girls had higher GC than did boys. Significant differences between the genders were found in body height and body weight among eight-year-old and nine-year-old children; in this case, boys were higher and heavier. In case of waist circumference, significant differences between boys and girls were found among eight-year-old, nine-year-old, ten-year-old, and eleven-year-old children. The higher values of waist circumference were determined in boys. In case of abdominal circumference, there was found just one significant difference between the genders, which was among ten-year-old children. Gluteal circumference significantly differed in six-year-old, seven-year-old, and eight-year-old children. The higher values were detected in girls. 

The gender- and age-specific WC values, AbC values, and GC values at selected percentiles are presented in Table 2, Table 3 and Table 4.

Female and male median WC percentiles (M) increase starting at the age of six. Both girls’ and boys’ median percentiles showed a continuous increase. We found similar trends regarding the median GC and AbC percentile curves. We saw a relatively constant increase in the median percentiles from ages 6 to 11 in both sexes.

**Figure 1:**
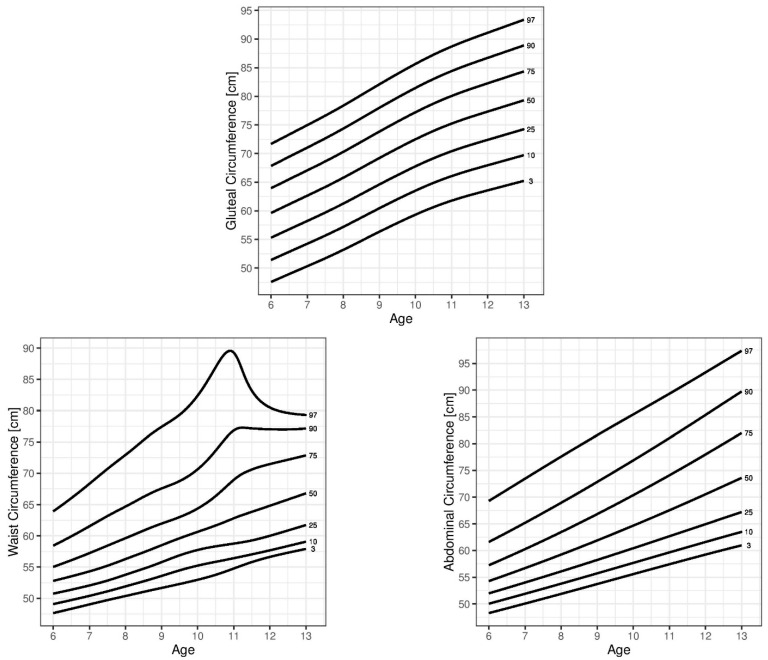
Percentile curves of the 3rd, 10th, 25th, 50th, 75th, 90th, and 95th percentiles for waist circumference (WC), abdominal circumference (AbC), and gluteal circumference (GC) by age in boys.

**Figure 2:**
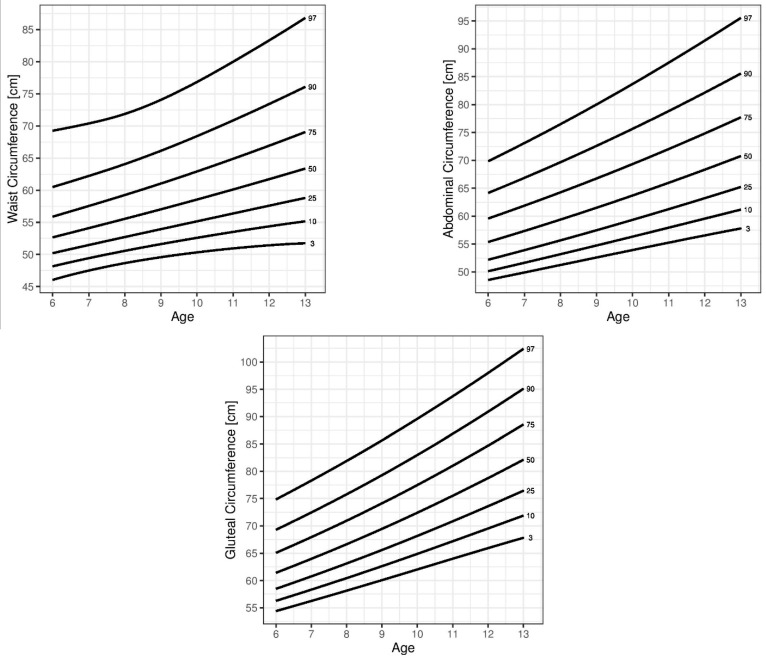
Percentile curves of the 3rd, 10th, 25th, 50th, 75th, 90th, and 95th percentiles for waist circumference (WC), abdominal circumference (AbC), and gluteal circumference (GC) by age in girls.

All percentile curves showed similar trends in both sexes, but the 90th and 97th WC percentiles in boys were exceptions: from the age of 10 they exceeded the values of the girls, reaching their peak at the age of 11 followed by a decrease in the case of the 97th percentile and a plateau in the case of the 90th percentile. The percentile curves generated for the WC, AbC, and GC for the 3rd, 10th, 25th, 50th, 75th, 90th, -and 97th percentiles are shown in Figure 1 (boys) and Figure 2 (girls).

## 4. Discussion

This study was conducted to provide age- and gender-specific percentile curves for anthropometric indicators for Czech children aged 6–11 years. Percentile data were developed from a sample of 2093 Czech elementary school children. The development of reference curve standards is usually used as a screening tool to identify childhood growth disorders [19]. These AbC and GC curves represent the attempt to update national reference curves. To the best of our knowledge, WC curves are the first attempt to develop percentile curves for this indicator. These percentile curves can be accepted as useful references for monitoring the adequate growth of Czech children, including their overweight and obesity levels. The distribution of body fat should also be taken into account when assessing individual’s body weight. The distribution of body fat, especially in terms of its uneven distribution, is a risk factor for health complications. An increase in circumferential parameters (WC, AbC) can indicate the development of abdominal obesity in early school-aged children. 

Circumference measurements are cheap and simple anthropometric methods, and both hip/gluteal circumference and waist circumference are good predictors of intra-abdominal fat and cardiovascular risk factors [20,21]. WC is considered the most accurate anthropometric method to predict metabolic syndrome (MetS) in children [22,23,24]. The hip/gluteal circumference measurement is a helpful tool to detect the presence of hypertension in children [12].

WC percentile references have been developed for children and adolescents living in different parts of world, such as Canada [25], the USA [26], Australia [27], the Netherlands [28], the United Kingdom [29], Germany [30], Turkey [31], Greece [32], Iran [33], Singapore [34], Brazil [4], and China [35]. Growth percentile charts of abdominal circumference and hip circumference have already been constructed in the Czech Republic. These charts were created on the basis of data obtained from the NAS in 1991 and 2001. Table 5 compares the average values of abdominal circumference and gluteal circumference within individual categories of children of early school age, obtained on the basis of relevant national surveys in 1991 and 2001 and our study. In the case of abdominal circumference, an upward trend in this somatic parameter is clearly visible, for both sexes. Gluteal circumference shows a different trend. In the case of boys, a smaller hip circumference is observed than in previous years; in the case of girls, we can talk about comparable values. The gluteal circumference of boys and girls from our research sample rather corresponds to the values in 1991. That would mean smaller development of gluteal circumference and, on the contrary, higher development of abdominal circumference in both genders with regard to both previous National Anthropological Surveys. This may suggest an increase in the amount of body fat in the abdominal area because we do not expect the development of muscles in the trunk or abdominal muscles in children at this age.

The Czech Republic was among the countries with a tradition of national anthropological surveys carried out every ten years [2]. Unfortunately, this tradition does not continue at present; it is being replaced by anthropological research of a regional nature. National growth charts are used in anthropometry, pediatrics, and other medical disciplines. Because of population trends, it would be desirable to update the Czech growth charts used so far.

Our study had several strengths, such as the fact that similar studies for developing percentile curves of WC have not been previously performed for Czech children. Furthermore, the anthropometric data were collected during a recent specific period of time, and we had a relatively large sample size of school-aged children from 6 to 11 years.

The limitations of this study include the fact that the choice of participating primary schools depended on their headmaster’s willingness to cooperate.

## 5. Conclusions

Our study provides WC, AbC, and GC percentile curves and reference values for Czech children aged 6–11 years. These percentiles could be used as a convenient, inexpensive, non-invasive, and quick tool to evaluate the growth status of children. They could be useful in medical practice and public health screening, as well as for an individual assessment of children’s growth. They could be used as a complementary screening tool for general overweight and abdominal obesity as well. Increased fat mass in the abdomen and hips could indicate the presence of metabolic risks and cardiovascular disease in adolescents and adults.

Given the detected changes in circumferential parameters compared to the results of previous Czech Anthropological Surveys, the use of these percentile charts is a suitable indicator for assessing increases in soft tissue in the abdomen and trunk as well as abdominal obesity in children.

## Figures and Tables

**Table 1:** Descriptive characteristics of observed anthropometric parameters and statistical significance between genders.

Age (years)	*n*	Height (cm)	Weight (kg)	WC (cm)	AbC (cm)	GC (cm)
Boys						
6	74	125.5 ± 6.1	24.9 ± 4.5	55.0 ± 4.5	57.5 ± 5.7	* 62.5 ± 5.6
7	223	132.1 ± 6.3	29.2 ± 5.4	56.2 ± 5.4	59.0 ± 6.1	* 63.8 ± 7.1
8	254	* 134.2 ± 5.7	* 30.6 ± 6.5	* 59.2 ± 6.6	62.3 ± 7.8	* 67.7 ± 6.6
9	179	* 139.0 ± 6.7	* 33.5 ± 7.0	* 61.0 ± 6.4	64.6 ± 7.5	70.8 ± 6.5
10	148	145.4 ± 7.5	38.0 ± 9.3	* 64.0 ± 8.1	* 68.6 ± 8.8	74.3 ± 7.7
11	130	148.3 ± 7.1	40.2 ± 8.4	* 65.1 ± 7.8	70.2 ± 9.1	76.4 ± 7.3
Girls						
6	95	124.0 ± 6.8	24.8 ± 6.1	55.5 ± 6.2	58.7 ± 7.0	* 65.1 ± 7.0
7	255	127.5 ± 6.4	26.0 ± 5.1	55.6 ± 5.7	59.4 ± 6.6	* 66.0 ± 5.9
8	251	* 132.1 ± 6.9	* 29.2 ± 5.7	* 57.7 ± 6.2	61.9 ± 7.1	* 68.9 ± 6.3
9	174	* 136.9 ± 7.0	* 31.6 ± 7.5	* 58.7 ± 7.0	63.3 ± 8.0	71.3 ± 7.6
10	177	144.6 ± 7.9	36.7 ± 8.6	* 61.0 ± 7.5	* 66.6 ± 8.5	75.5 ± 7.6
11	133	147.0 ± 7.4	39.0 ± 9.6	* 62.1 ± 8.0	68.5 ± 9.6	77.7 ± 8.7

Note: mean ± standard error. AbC, abdominal circumference; GC, gluteal circumference; WC, waist circumference. * *p* ≤ 0.05.

**Table 2:** Tabulated waist circumference (WC) percentile values by age.

Percentile							
Age (Years)	3	10	25	50	75	90	97
Girls							
6	46.0	48.2	50.2	52.7	55.9	60.5	69.3
7	47.5	49.4	51.5	54.1	57.5	62.2	70.4
8	48.7	50.6	52.7	55.5	59.3	64.1	71.9
9	49.6	51.6	53.9	57.0	61.0	66.1	74.1
10	50.3	52.6	55.2	58.6	62.9	68.4	76.9
11	50.9	53.5	56.4	60.1	64.9	70.9	80.0
Boys							
6	47.7	49.1	50.8	52.8	55.0	58.4	63.9
7	49.0	50.4	52.1	54.3	57.2	61.5	68.3
8	50.4	51.9	53.8	56.3	59.6	64.7	72.9
9	51.7	53.6	55.8	58.5	62.0	67.6	77.4
10	53.0	55.2	57.7	60.6	64.4	70.6	82.4
11	54.8	56.4	58.8	62.8	68.9	76.8	89.3

**Table 3:** Tabulated abdominal circumference (AbC) percentile values by age.

Percentile							
Age (Years)	3	10	25	50	75	90	97
Girls							
6	48.6	50.1	52.2	55.4	59.6	64.2	69.9
7	49.9	51.6	53.9	57.3	61.9	66.9	73.1
8	51.2	53.2	55.7	59.4	64.3	69.7	76.5
9	52.6	54.7	57.5	61.5	66.8	72.6	80.0
10	53.9	56.3	59.3	63.7	69.3	75.7	83.7
11	55.2	57.9	61.3	66.0	72.0	78.8	87.5
Boys							
6	48.3	50.1	52.0	54.3	57.3	61.6	69.3
7	50.1	51.9	54.0	56.7	60.3	65.2	73.5
8	51.9	53.8	56.1	59.2	63.5	68.9	77.6
9	53.7	55.7	58.2	61.9	66.8	72.8	81.6
10	55.6	57.7	60.4	64.7	70.4	76.9	85.5
11	57.4	59.7	62.7	67.5	74.1	81.0	89.3

**Table 4:** Tabulated gluteal circumference (GC) percentile values by age.

Percentile							
Age (Years)	3	10	25	50	75	90	97
Girls							
6	54.4	56.3	58.5	61.4	65.1	69.3	74.9
7	56.2	58.3	60.8	64.0	67.9	72.5	78.3
8	58.1	60.4	63.1	66.6	71.0	75.8	81.9
9	60.1	62.6	65.6	69.5	74.1	79.3	85.7
10	62.0	64.9	68.2	72.4	77.5	83.0	89.6
11	64.0	67.2	70.8	75.5	81.0	86.9	93.7
Boys							
6	47.6	51.4	55.3	59.6	64.0	67.8	71.7
7	50.3	54.2	58.2	62.6	67.1	71.0	75.0
8	53.2	57.2	61.3	65.8	70.3	74.4	78.4
9	56.3	60.4	64.6	69.2	73.8	78.0	82.1
10	59.3	63.5	67.8	72.5	77.2	81.5	85.7
11	61.8	66.1	70.4	75.2	80.1	84.4	88.7

**Table 5:** Comparison of abdominal circumference (AbC) and gluteal circumference (GC) of Czech children in 1991, 2001 (NAS), and 2020 (our study).

Age (Years)	AbC (cm)			GC (cm)		
Boys	2020	2001	1991	2020	2001	1991
6	57.5 ± 5.7	56.8 ± 5.4	56.0 ± 4.7	62.5 ± 5.6	65.1 ± 5.6	63.7 ± 5.0
7	59.0 ± 6.1	58.4 ± 5.6	57.3 ± 4.8	63.8 ± 7.1	67.5 ± 6.0	65.7 ± 5.2
8	62.3 ± 7.8	60.7 ± 6.3	59.1 ± 5.3	67.7 ± 6.6	70.9 ± 6.3	68.4 ± 5.5
9	64.6 ± 7.5	62.5 ± 7.5	61.3 ± 6.3	70.8 ± 6.5	73.2 ± 7.2	71.4 ± 6.3
10	68.6 ± 8.8	64.9 ± 7.8	63.3 ± 7.1	74.3 ± 7.7	76.3 ± 7.2	74.1 ± 6.9
11	70.2 ± 9.1	66.9 ± 8.3	65.3 ± 7.4	76.4 ± 7.3	78.8 ± 7.9	76.7 ± 7.1
Girls						
6	58.7 ± 7.0	56.2 ± 5.8	54.7 ± 4.8	65.1 ± 7.0	65.3 ± 5.8	64.0 ± 5.0
7	59.4 ± 6.6	57.5 ± 5.9	56.2 ± 5.4	66.0 ± 5.9	67.9 ± 6.0	66.6 ± 5.7
8	61.9 ± 7.1	59.2 ± 6.5	57.9 ± 5.7	68.9 ± 6.3	70.8 ± 6.5	69.3 ± 5.9
9	63.3 ± 8.0	61.2 ± 6.9	60.0 ± 6.8	71.3 ± 7.6	73.6 ± 6.8	72.5 ± 6.8
10	66.6 ± 8.5	63.4 ± 7.6	61.3 ± 6.9	75.5 ± 7.6	77.0 ± 7.1	75.2 ± 6.9
11	68.5 ± 9.6	65.2 ± 8.2	64.1 ± 7.4	77.7 ± 8.7	80.5 ± 8.0	79.0 ± 7.4

## Data Availability

All data generated or analyzed during this study are included in this published article. The datasets used and/or analyzed during the current study are available from the corresponding author on reasonable request.

## Abbreviations

WC: waist circumference; AbC: abdominal circumference; GC: gluteal circumference; M: median; SD: standard deviation; BMI: body mass index; BIA: bioelectrical impedance analysis; NAS: National Anthropological Survey.
